# Supplementary material for: Interleukin-3-Receptor-α in Triple-Negative Breast Cancer (TNBC): An Additional Novel Biomarker of TNBC Aggressiveness and a Therapeutic Target
Source: Cancers (Basel). 2022 Aug 13;14(16):3918. doi: 10.3390/cancers14163918 (PMC9406043; doi:10.3390/cancers14163918)
Supplement: Supplementary file 1 [file cancers-14-03918-s001.zip › cancers-1792283-supplementary.pdf]

Interleukin-3-Receptor-α in Triple-Negative Breast Cancer (TNBC): An Additional Novel Biomarker of TNBC

Aggressiveness and a Therapeutic Target

Malvina Koni, Isabella Castellano, Emilio Venturelli, Alessandro Sarcinella, Tatiana Lopatina, Cristina Grange, Massimo Cedrino, Saveria Femminò, Paolo Cossu-Rocca, Sandra Orrù, Fabrizio D'Ascenzo, Ilaria Cotellessa, Cristian Tampieri, Carla Debernardi, Giovanni Cugliari, Giuseppe Matullo, Giovanni Camussi, Maria Rosaria De Miglio, and Maria Felice Brizzi

Supplementary Information

Materials and Methods

Cell lines

The following TNBC cell lines were used: MDA-MB-231, MDA-MB-453, MDA-MB-436, MDA-MB-157, BT-549, HCC-1395, and Hs-578T. The pathological features of each cell line are reported in Supplementary Table S1; the MCF10A cell line, a non-neoplastic breast cancer cell subtype, was cultured as indicated by the manufacturer and served as a negative control. All cell lines were provided by the ATCC (Manassas, VA) and cultured as indicated by the manufacturers. The M07 leukaemic cells were established in the lab (49). MDA-MB-231 cells were cultured in a DMEM medium, supplemented with 10% FBS. Hs-578T cells were cultured in a DMEM medium, supplemented with 10% FBS and 0.01 mg/ml insulin. MDA-MB-157, MDA-MB-453, and HCC-1395 cells were cultured in an RPMI medium, supplemented with 10% FBS. BT-549 cells were cultured in an RPMI medium, supplemented with 10% FBS and 0.023 U/ml insulin. MDA-MB-436 cells were cultured in an RPMI medium, supplemented with 10% FBS, 16 µg/ml glutathione, and 10 µg/ml insulin. Culture media and FBS were from Euroclone S.p.A. (Pero, MI, Italy) and Invitrogen (Carlsbad, CA, USA) respectively. Insulin was from Invitrogen (Carlsbad, CA, USA). All culture media were supplemented with 1% Pen/Strep from Invitrogen (Carlsbad, CA, USA).

Western blot analysis

Cells were lysed using a RIPA buffer (50 mM Tris (pH7.5), 150 mM NaCl, 1% Triton X100, 1% Na deoxycholate, 0.1% SDS and protease inhibitors). Cell lysates were centrifuged at 13000 rpm for 20 min, and the supernatants were collected and assayed for protein concentration using a PierceBCA protein assay kit (Thermo Scientific, Rockford, IL, USA). Proteins were run on SDS–PAGE under reducing conditions. Following SDS–PAGE, proteins were transferred to Nitrocellulose membranes (Bio-Rad, Hercules, CA, USA), saturated with 5% BSA for 2 h, incubated with specific antibodies, and then detected with peroxidase-conjugated secondary antibodies and the chemiluminescent ECL reagent (Bio-Rad, Hercules, CA, USA). Protein expression was evaluated using ChemiDoc XRS+System (Bio-Rad). The following antibodies were used: anti-Vimentin (Abcam #ab8978), anti-N-Cadherin (Abcam #ab18203), anti-GAPDH (Abcam # ab8245), anti-β-Actin (Abcam #ab8227), anti-CD31 (Abcam #ab28364), and anti-IL-3Rα/CD123 (R&D Systems #MAB301-100). The secondary antibodies conjugated with peroxidase were purchased from Cell Signalling Technologies.

FACS Analysis

For the FACS analysis of IL-3Rα/CD123 surface marker, MDA-MB-231, MDA-MB-453, MDA-MB-436, MDA-MB-157, BT-549, Hs-578T, HCC-1395, MCF10A, and M07 cells were harvested and subsequently disaggregated using non-enzymatic dissociation and washed in PBS. Cells were

then stained with human anti-CD123 antibody for 30 min. Flow cytometric analysis was performed using a Cytoflex flow cytometer (Beckman Coulter, Brea, CA, United States) equipped with the CytExpert software version 2.3.0.84. During acquisition, the median fluorescence intensity (MFI) was corrected for medium background and gated based on their respective fluorescence intensity as per the manufacturer's instructions.

Real-time PCR analysis

Real-time polymerase chain reaction (PCR) was performed to detect SLUG, ZEB 1, and TWIST transcript in Hs-578T, HCC-1395, MDA-MB-231, and MDA-MB-436 cell lines untreated or treated with IL-3 (5 ng/ml of recombinant human IL-3) (BD Biosciences, San Jose, CA, USA) for 24 h. The total RNA was extracted using an RNeasy Kit (Qiagen). RNA was reverse-transcribed using a High Capacity cDNA Reverse Transcription Kit (Thermo Fisher Scientific) and subjected to quantitative real-time PCR (qRT–PCR) using QuantStudio12KFlex (Applied Biosystem). The β-actin served to normalise mRNA expression.
Primer sequences: TWIST F: 5'-GTCCGCAGTCTTACGAGGAG-3'
R: 5'-GCTTGAGGGTCTGAATCTTGCT-3'
SLUG F: 5'-CGAACTGGACACACATACAGTG-3'
R:5'-CTGAGGATCTCTGGTTGTGGT-3'
β-ACTIN F: 5'-TGAAGATCAAGATCATTGCTCCTC-3'
R:5'-CACATCTGCTGGAAGGTGGAC-3'
ZEB1 F: 5'GAAGAGATCAAAGACATGTGACGC-3'
R-5'TCTCCACTGTGAATTCTTAAGTGCTC-3'

Tube-like structure formation assay

Cells were non-enzymatically detached with Cell Dissociation Solution Non-enzymatic 1x (Thermo Scientific, Waltham, MA, USA), counted, washed to remove the serum, and seeded onto a thick (50 µl) layer of growth factor reduced Matrigel (BD Biosciences, San Jose, CA, USA) in a 24-well plate (Corning). Cells were resuspended in 500 µl of serum-free DMEM supplemented with saline (Ctrl), 1 ng/ml, or 5 ng/ml of recombinant human IL-3 (BD Biosciences, San Jose, CA, USA) (3×10^4 cells/well) as indicated. After 24 h, tube-like structures were observed and counted with a Nikon-inverted microscope (20X), and a Leica-digital camera was used. Three different fields/well were counted by two blinded independent operators (each experiment was performed in triplicate).

Bioinformatics analysis

Using The Cancer Genome Atlas (TCGA), limited to the BRCA data, the datasets were downloaded from the TCGA website (https//tcga data.nci.nih.gov, accessed on 14 November 2021) for gene expression analyses. These data are publicly accessible, and there was no further ethical approval required from the Ethics Committee. Overall, 1,205 breast cancer samples from TCGA were investigated to explore the relationship between IL-3Rα gene expression and the aggressiveness of the disease. The mRNA expression data were grouped into 1,080 luminal breast cancer samples and 125 TNBCs, using the PAM50 Subtype classification provided by TCGA. For differential expression analyses, we used DESeq2, an R Bioconductor package using a model based on the negative binomial distribution. Gene counts input into DESeq2 were rounded to the nearest whole number, normalised, and filtered for a minimum of 10 counts per gene in each sample.

Cell line	Subtype	Source	Tumor type
MDA-MB-231	Basal B	Pleural Effusion	Metastatic Adenocarcinoma
MDA-MB-436	Basal B	Pleural Effusion	Adenocarcinoma
MDA-MB-453	Luminal	Pleural Effusion	Metastatic Carcinoma
MDA-MB-157	Basal B	Pleural Effusion	Medullary Carcinoma
Hs-578T	Basal B	Primary Tumor	Carcinoma Sarcoma
HCC-1395	Basal B	Primary Tumor	Ductal Carcinoma
BT-549	Basal B	Primary Tumor	Invasive Ductal Carcinoma

Supplementary Table S1. Clinical and pathological features of different TNBC cell lines expressing the IL-3Rα.

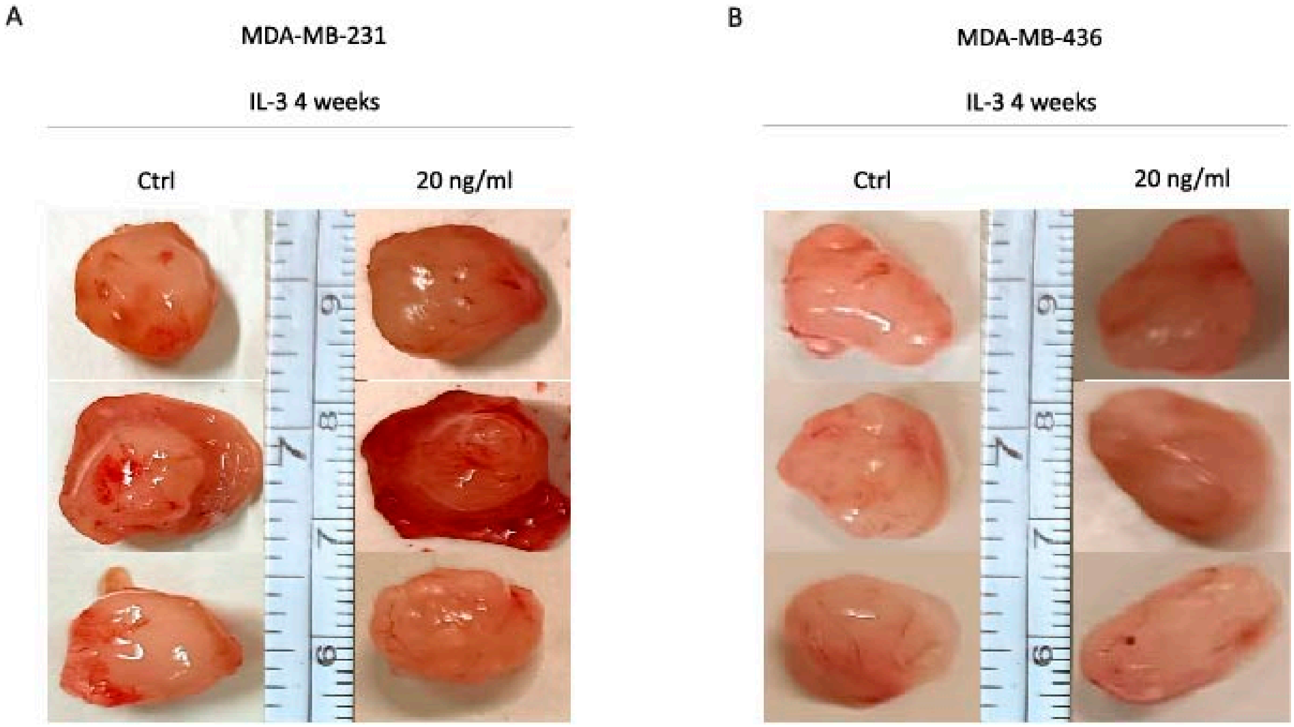

Supplementary Figure S1. Images refer to primary tumours of mice injected with MDA-MB-231 cells **(A)** and MDA-MB-436 **(B)** untreated or treated with IL-3 (20 ng/ml).

Figure 3A

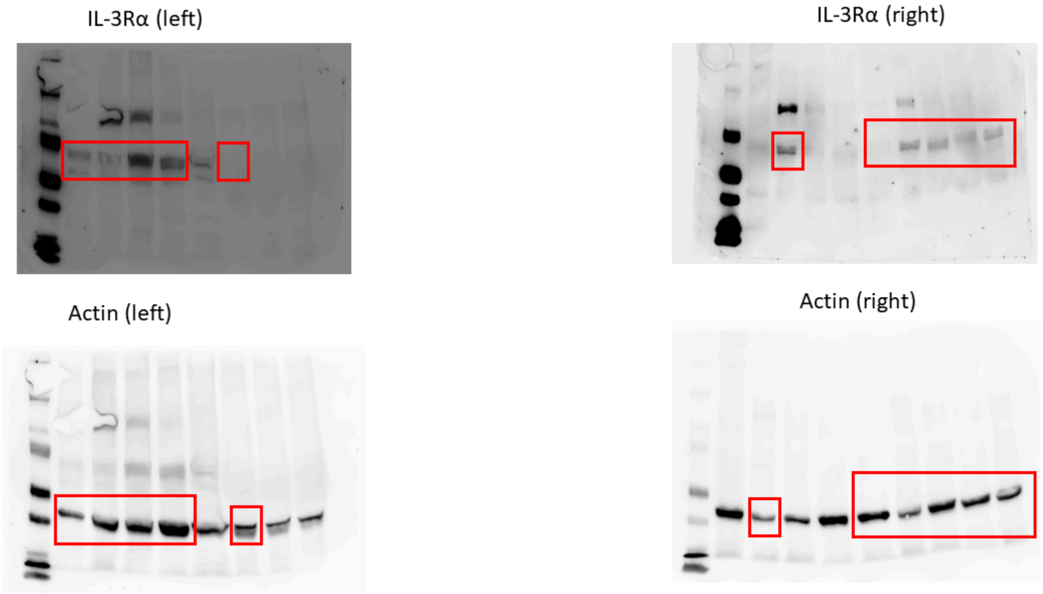

Figure S2. Full pictures of the Western blots for Figure 3A.

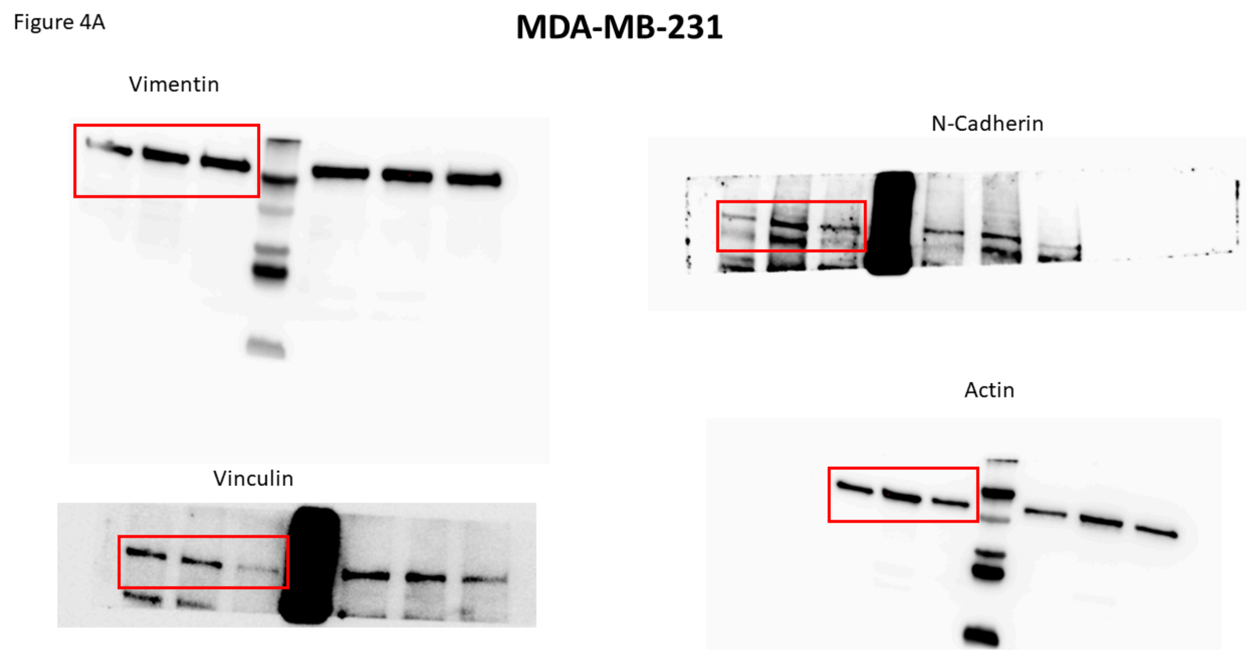
Figure 4A
MDA-MB-231
Vimentin
N-Cadherin
Actin
Vinculin

(A)

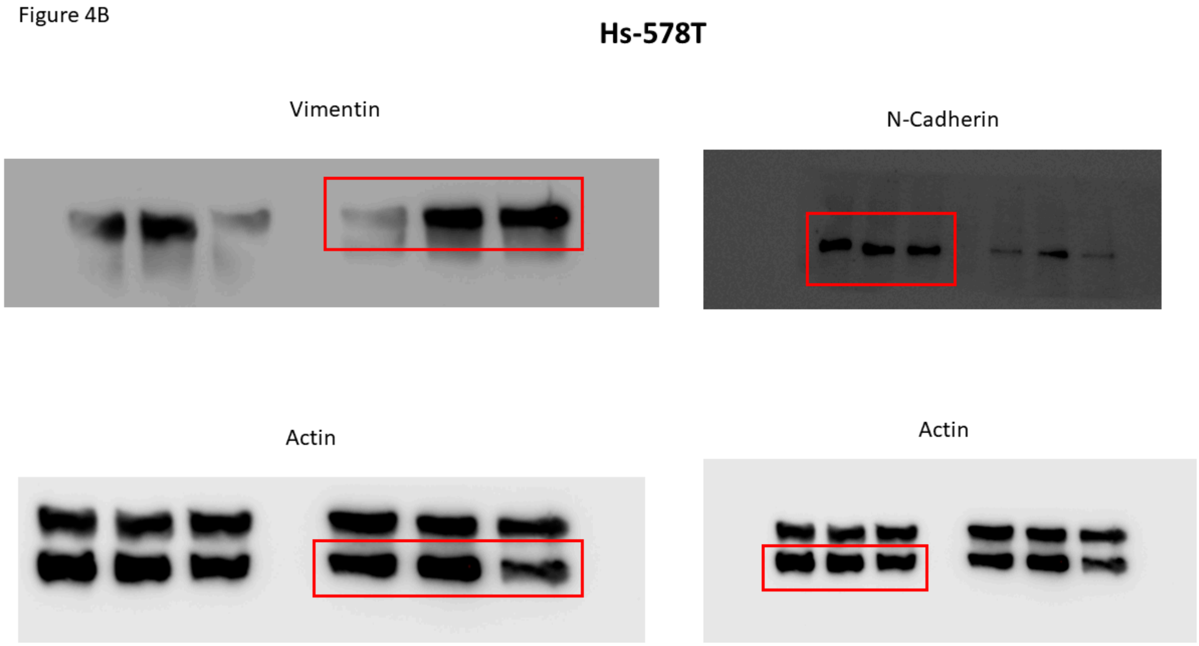
Figure 4B
Hs-578T
Vimentin
N-Cadherin
Actin
Actin

(B)

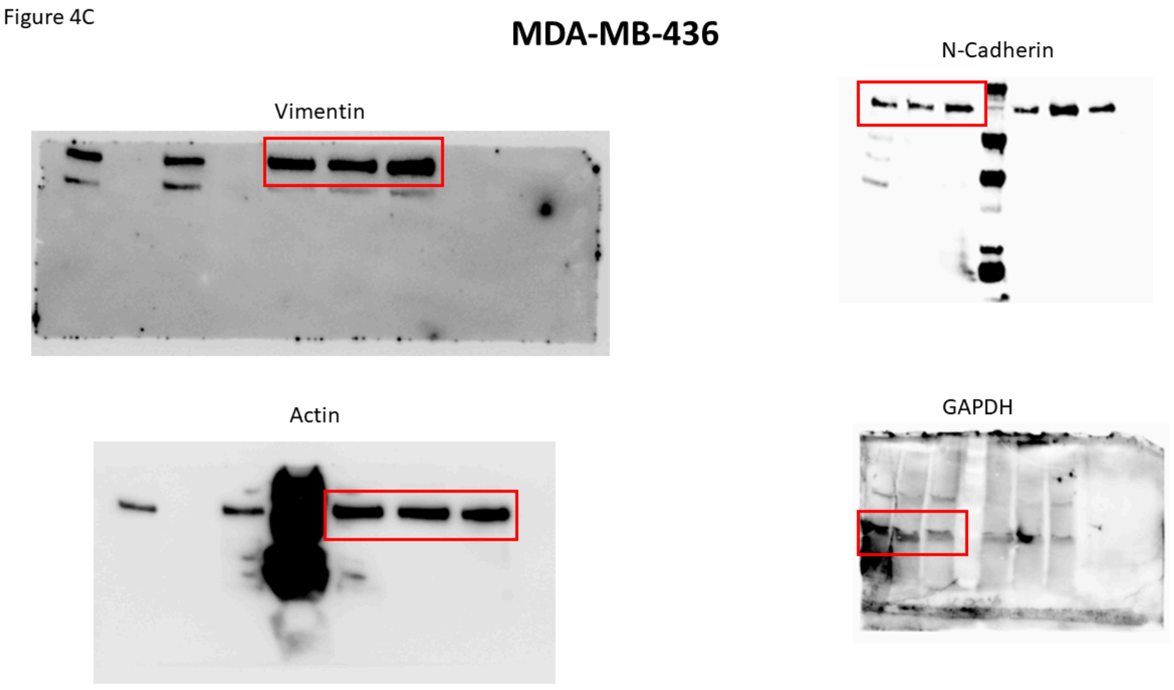

(C)

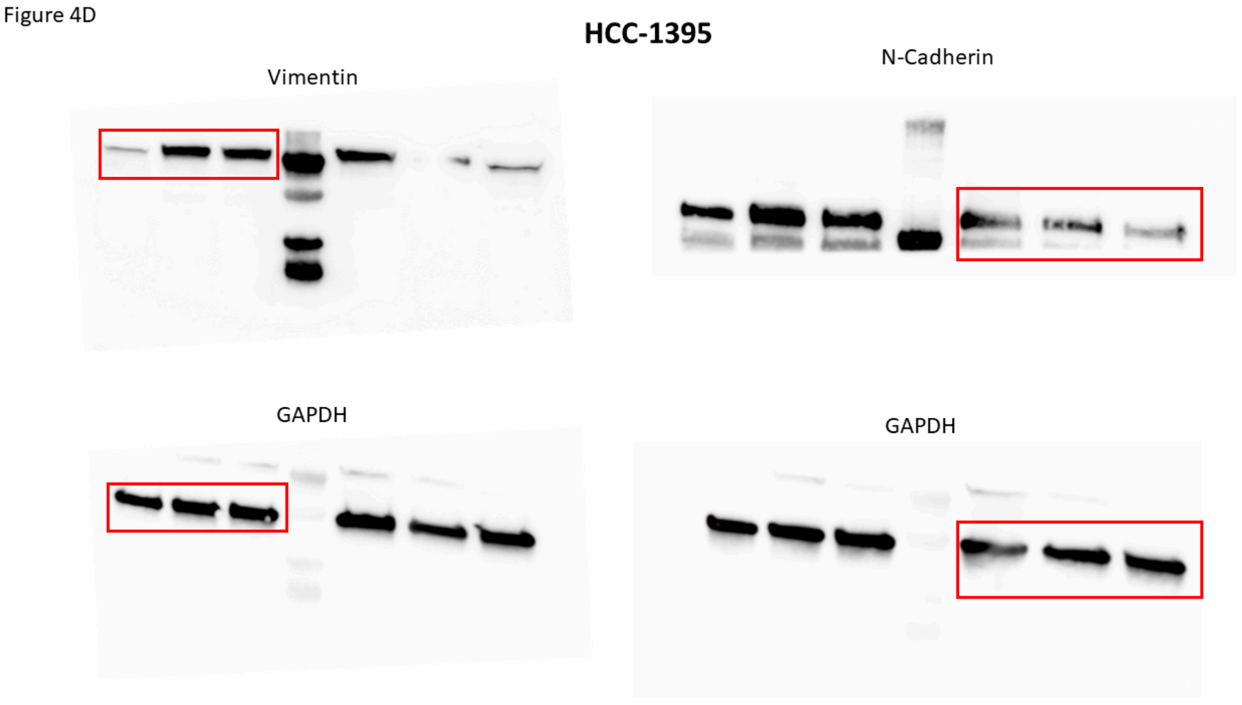

(D)

Figure S3. Full pictures of the Western blots for Figure 4A–D.
